# Advancing Detection of Crime Scene Staging in Intimate Partner Homicide: A Study in Gauteng, South Africa

**DOI:** 10.1177/10778012251369027

**Published:** 2025-08-19

**Authors:** Naomi Griffiths, James Ojochenemi David, Sekgololo Angel Mabudusha

**Affiliations:** 1 56866 UNISA, Pretoria, South Africa

**Keywords:** crime scene staging, intimate partner homicide, Gauteng, offender profiling, rational choice theory

## Abstract

Crime scene staging, though underexplored in South Africa, gained attention following the Jason Rohde case involving the staged murder of his wife. This study examines the prevalence and dynamics of staging in intimate partner homicides, particularly in Gauteng. Eighteen investigating officers from diverse police stations were interviewed to explore their experiences. Findings revealed parallels with international research, with analysis of modus operandi, victimology, and offender profiles. These were assessed using rational choice theory to understand motivations and improve detection. The study concludes with a proposed 10-point plan, integrating local insights and global strategies to enhance investigative approaches to crime scene staging.

## Introduction

Alexander Pope once noted, “He who tells a lie is not sensible of how great a task he undertakes; for he must be forced to invent twenty more to maintain that one” (Pope, 1737). Lies have woven through the fabric of humanity since ancient times, from the deceit of Cain to the staged scene of Joseph's apparent demise. In contemporary society, the correlation between deception and criminal behavior is documented. As Azizli et al. (2016, p. 34) assert, deceptive individuals often find themselves entangled in misconduct or crime.

Consequently, an analysis of a nation's crime rate becomes a reflection of its prevalence of deceitful individuals. South Africa, plagued by a disturbingly high rate of violent crimes, particularly intimate partner murders, stands as a poignant example. The trajectory of intimate partner violence gained specific attention in 2008 during a conference themed “Gender Violence” by the Centre for the Study of Violence and Reconciliation (Bezuidenhout, 2023, p. 279). Since then, gender-based violence, including its most extreme manifestation in intimate partner homicides, has remained a focal point for law enforcement, notably the South African Police Service (SAPS). The government's response has materialized in initiatives like the annual “16 Days of Activism” campaign, spanning from 25 November to 10 December. Tragically, South Africa still grapples with an alarmingly high rate of intimate partner homicides, with statistics revealing a harrowing reality: a woman is killed by her intimate partner every 8 hr, amounting to an estimated four deaths per day and 1,460 per year (Abrahams et al., 2013, p. 1).

Despite governmental efforts, the prevalence of such crimes persists, transcending boundaries of gender, age, socioeconomic status, religion, or sexual orientation. The case of Susan Rohde, whose body was discovered under suspicious circumstances at the Spier Wine Estate, serves as a distressing illustration. Initially deemed a suicide, meticulous investigation revealed the truth: Susan was a victim of intimate partner homicide, with her husband, Jason Rohde, orchestrating a staged scene in a bid to obfuscate his guilt (State v Rohde, 2019). This case underscores the insidious nature of crime scene staging, a tactic employed by offenders to evade justice. Crime scene staging, or detection avoidance as described by Ferguson (2021) is not a modern phenomenon; it echoes ancient tales of deception, akin to Joseph's brothers fabricating his demise in Genesis. Consequently, the first work on the subject is a century old (Gross, 1924).

Jason Rohde sought to weave a narrative of deceit, but forensic scrutiny dismantled his facade, leading to his conviction and sentencing. The Rohde case catalyzed a deeper inquiry into the prevalence and detection of staged scenes in intimate partner murders, particularly in the Gauteng province. The province, which is South Africa's most populous and economically significant region, is home to major cities such as Johannesburg and Pretoria. Gauteng's high levels of violent crime render it a critical site for analyzing femicide and crime scene staging. In the third quarter of 2022, Gauteng recorded 1,721 murders—representing the second-highest provincial total in the country and a 9.6% increase from the same quarter in the previous year (SAPS, 2023).

Johannesburg, situated in the southern part of the province, ranks highest nationally in contact crimes such as murder, attempted murder, and assault. Notably, five of the 10 police stations reporting the highest number of murders nationwide are located in Gauteng, including Johannesburg Central, Alexandra, Tembisa, Temba, and Hillbrow. For example, Johannesburg Central alone recorded 1,156 contact crimes in the final quarter of 2022. These statistics underscore the region's prominence in national crime trends and its relevance for femicide research.

Further analysis of causative factors for murder in the province reveals that 403 cases were linked to robbery, 44 to hijacking, 28 to rape-related incidents, and 37 to other crimes (SAPS, 2023). Although current data on domestic violence-related murders remains unclear, earlier reports suggest that an overwhelming majority of murders occur between individuals who are known to each other. According to Bezuidenhout (2023, p. 183), using SAPS data from 2009/2010, approximately 70% to 80% of all murders involve acquaintances, most commonly intimate or former partners. This pattern remains a crucial consideration in femicide investigations. However, caution is warranted in interpreting crime statistics, as underreporting remains a significant challenge in the South African context (Bezuidenhout, 2023, p. 92). Essentially, the province’s staggering murder rate prompts critical questions about the accurate classification and detection of such crimes (SAPS, 2023), and whether these can be improved. While official statistics shed light on the landscape of crime, the true extent of the problem remains elusive, with many cases potentially misclassified or overlooked.

Inquests, governed by the Inquest Act (Act 58 of 1959), offer a legal avenue for probing suspicious deaths, yet they too may fail to unearth the truth, as evidenced in the Rohde case (State v Rohde, 2019). The misclassification of deaths, especially those stemming from assault, underscores the urgent need for more rigorous investigative protocols. Dayan and Bitton (2023) underscore this imperative, suggesting that improved tools and criteria could mitigate misidentifications and enhance justice delivery. Against this backdrop, a qualitative study was conducted to investigate the phenomenon of crime scene staging in intimate partner homicides within Gauteng, by exploring how law enforcement officials understand, detect, and respond to such cases, and to evaluate how the rational choice theory can explain offender behavior with the view to guide improved investigative practices. By delving into concluded intimate partner homicide cases and probing law enforcement's understanding of staging, the study aimed to identify elements specific to the South African landscape and to assess whether or not they are similar to international findings. In doing so, strategies drawn from international studies—combined with the insights of investigating officers experienced in such cases—can be used to formulate practical guidelines for more effective detection. This, in turn, could strengthen the criminal justice system's ability to deliver justice while reducing the risk of civil claims against law enforcement agencies. In essence, this study serves as a clarion call for enhanced training and awareness within institutions like the SAPS, paving the way for more robust investigative practices and, ultimately, a safer society for all South Africans.

### Conceptualizing Crime Scene Staging

Chancellor and Graham (2014, p. 19) have observed that although most forensic practitioners and police officials encounter staged crime scenes in their careers, there is a glaring lack of statistical data to gauge the prevalence of such incidents. The true scope of staging remains elusive, as some cases may never come to light due to the adeptness of the perpetrator in executing sophisticated deceptions (Ferguson, 2021, p. 2). Complicating matters further are factors like the underreporting of staging within murder cases and the classification of deaths as undetermined during postmortem examinations. While several studies have delved into crime scene staging internationally, literature on the subject remains sparse compared to other criminological topics (Ferguson, 2021, p. 2). Eke (2007) conducted the pioneering comprehensive study on staging, followed by notable works by Hazelwood and Napier (2004), Keppel and Weis (2004), Schlesinger et al. (2012), and Pettler (2015). Ferguson (2015) conducted research that focused specifically on staged suicides.

Previous research has yielded varied findings regarding the types of crimes murders are staged as. Turvey (2000) discovered that domestic murders are often disguised as stranger-burglaries, while Hazelwood and Napier (2004, p. 752) found staging to encompass various crimes such as sexual murders, home-invasion murders, and autoerotic fatalities. Schlesinger et al. (2012, p. 46) identified arson as the most prevalent staging method, followed by burglary/robbery and accidents. Ferguson (2021) explains that detection avoidance differs from crime scene staging in that the former typically involves attempts to destroy or eliminate physical evidence, whereas staging almost always includes a deliberate attempt to mislead investigators through a fabricated account or false testimony provided to the police. A Queensland, Australia based study found that some form of detection avoidance behavior was noted in 43% of intimate partner femicide cases (McLachlan & Ferguson, 2024, p. 11). The perpetrators of staged murders typically exhibit ordinary appearances, often defying stereotypes associated with violent offenders. Ferguson and Petherick (2016, p. 14) found that most offenders were males, with none of the offenders in their study belonging to law enforcement. Pettler (2015, p. 72) observed that offenders are often male, unemployed, and may struggle with substance abuse. Ferguson (2021) indicates that a definitive typology is elusive, as many of these staged homicides occur within intimate relationships.

However, Pettler (2015, pp. 90–95) identified six typologies of crime scene stagers, ranging from “The Cleaner” who seeks to eliminate evidence, to “The Planner” who meticulously orchestrates the crime and subsequent staging. Victimology plays a crucial role in understanding staged crime scenes, with Hazelwood and Napier (2004, p. 757), highlighting inconsistencies often found in victims themselves. Ferguson (2021, p. 9) noted that most victims are young adult females, primarily discovered in their personal spaces, such as bedrooms, often shared with an intimate partner. In cases staged as suicides, victims are typically young adult females, underscoring the importance of victim demographics in understanding staging incidents (Ferguson, 2021, p. 9). Pettler (2015, p. 108) too emphasizes that females are most often the victims of staged crime scenes.

### Femicide in South Africa

Brodie et al. (2023, p. 16) define femicide as the killing or murder of a woman. The term itself derives from the Latin words “femina” (woman) and “cide” (killing). Mobayed Vega (2024, p. 2) defines femicide as the gender-related killing of women and girls. Florence et al. (2022, p. 295) state that the term femicide refers to the killing of a female because of abusive and violent acts by a male and can also include an intimate partner. While many cases of femicide involve women being killed by their current or former partners, femicide also includes other types of killings—like those related to sexual violence, so-called “honor” crimes, dowry disputes, or other reasons rooted in hatred or discrimination against women.

According to data from the SAPS website, the murder rate of women witnessed a notable increase from 2020 to 2022, with a 27% rise during this period (SAPS, 2023). This study focuses on Gauteng, which is the smallest of South Africa's nine provinces by land area, covering approximately 18,178 km^2^ and located in the northeastern part of the country. It is the most densely populated and economically significant region of South Africa. It is home to major metropolitan areas such as Johannesburg, which is the country's largest city, and Pretoria, which is the administrative capital. Gauteng serves as a national hub for finance, commerce, and industry. Gauteng has some of the highest levels of violent crime in the country, making it an important place to study femicide and how crime scenes are sometimes staged in cases where women are killed by their partners. The victims in this study, all females, correlating with findings from international studies pertaining to crime scene staging, were all killed by males. For this reason, the killings could be classified as femicides.

Abrahams et al. (2013) conducted a study comparing femicide rates from 1999 to 2009, during which they observed a decrease in the overall murder rate of women, from 3,793 in 1999 to 2,363 in 2009. However, the latest statistics from the SAPS website reveal a concerning trend, with 3,843 women murdered in 2022 alone. Despite previous declines, it appears that the murder rate of women is once again on the rise (SAPS, 2023). Abrahams et al. (2013) noted a parallel decrease in intimate partner femicide alongside the general decline in femicide. This suggests a linear relationship between these two phenomena. If this relationship persists, it implies that intimate partner femicide is also increasing, mirroring the upward trend in the overall murder rate of females. Brodie et al. (2023, p. 82) emphasize that although intimate femicide accounts for most reported cases in which women are victims, it represents only around 30% of all female homicides covered in the media—suggesting that many such cases may be inaccurately represented or underreported. Furthermore, Abrahams et al. (2013, p. 3) discovered that despite the overall decrease in murders of women, intimate femicide still accounts for the highest proportion (56%) of such cases, with South Africa's intimate femicide rate being twice that of the United States. A review on gender-based violence by the Centre for the Study of Violence and Reconciliation (CSVR, 2016, p. 6) revealed alarming statistics, indicating that more than 50% of women in Gauteng have experienced intimate partner violence, which often leads to intimate femicide.

Turvey (2000) discovered in his research that staging often serves as a tactic to obscure the intimate connection between the perpetrator and the victim. Pettler (2015, p. 82) underscores the significance of interpersonal relationships in the examination of crime scene staging. Hazelwood and Napier (2004, p. 758) observed that 85% of fatalities in their study involved intimate partner violence. Falsifying the narrative by these offenders was mostly observed by stating another attacker was present and that they were a covictim or protector (McLachlan & Ferguson, 2024, p. 9). Schlesinger et al. (2012, p. 46) reported that 18.84% of cases involved intimate partner murders, with staging methods encompassing verbal staging (28.84%), accidents (19.23%), burglary/robbery (19.23%), arson (11.53%), suicide (7.69%), and murder-suicide (7.69%). Similarly, Ferguson (2021, p. 9) states that intimate partner relationships are prevalent in most cases they examined. Dayan and Bitton (2022, p. 20) pointed out that staging was particularly widespread in murders where the victim and perpetrator had a relationship or were acquainted, thus underscoring the prevalence of staging in intimate partner murders. Ferguson and McLachlan (2023, p. 354) suggest that the concealment by the perpetrators is an extension of the coercive control some intimate partners hold over their female counterparts, and that they are seen as expert abusers with clear rationality.

### Theory and Crime Scene Staging

Some crimes are impulsive in nature, while others are meticulously planned to avoid detection. The calculated actions involve a rational decision by weighing up the costs and benefits of a certain action (Bezuidenhout, 2023, p. 143). The rational choice theory from the classical school of thought encompasses precisely this rational decision-making process, driven by a cost-benefit analysis of the situation. The essential goal is utility maximization, as the individual decides on the choice of action that would maximize benefits or minimize costs. The decision is shaped by human actions and thought processes, influenced by social relationships and individual proficiencies (Bezuidenhout, 2023, p. 144; Houck & Siegel, 2010).

Morgan et al. (2012, p. 52) suggest that offenders are not rational in every part of their being. They may, in fact, progress through everyday life filled with uncertainty and risks, and satisfactory rewards, often not achieved. However, when it comes to the crimes they engage in, informed decisions weighing up every detail of the risk and reward (Morgan et al., 2012, p. 53). Brown et al. (2010, pp. 171–172) state that the rational choice extends just beyond whether to commit a crime to the type of crime, modus operandi, and victim selection. In crime scene staging, this rational thought process extends to the staging behavior after the initial crime, to further minimize the risk of being apprehended. The type of crime, that is, murder and the victim selection (personal) appear to be a rational decision and as stated by Siegel (2001, p. 106): even serial murderers, who appear to be the irrational of all, carefully select victims based on rational choice processes. In the response to crime, Morgan et al. (2012, p. 53) suggested that target hardening, increased risk for committing crime, and a reduction in the rewards associated with crime are valuable factors to consider.

### The Detection of Crime Scene Staging

At the core of crime scene staging lies the fundamental concept of deception (Ferguson, 2021, p. 2). Essentially uncovering why people lie, how they lie and how these lies can be uncovered, can provide investigators with invaluable insights into the detection of crime scene staging (Ferguson, 2021). Shaw et al. (2013) revealed that providing professionals with deception-specific training significantly enhances their ability to detect lies.

In the investigation of a murder case, the investigating officer employs a range of tools and expertise. While the crime scene offers valuable insights, input from various stakeholders such as forensic pathologists, forensic experts, criminologists, witnesses, and suspects is indispensable in unraveling the intricacies of the crime (Pettler, 2015). Ferguson (2021, p. 3) notes that the ability to identify staged behaviors during the investigation process varies depending on the type of investigator involved, whether it be a detective, criminologist, forensic pathologist, or other forensic expert. Schlesinger et al. (2012, p. 50) recommend that law enforcement officials and academia engage convicted crime scene stagers to gain insight into their motives for engaging in staging behavior.

### The Role of the Police

Abrahams et al. (2013, p. 4) highlighted a concerning lack of improvement in the investigation of murders involving female victims from 1999 to 2009. They underscored the inadequacy of case investigations, citing statistics that point to persistent shortcomings. Despite the century-long presence of staging and deception in criminal investigations, the efficacy of various investigative strategies and techniques remains uncertain due to limited research and the absence of error rate indicators (Chancellor & Graham, 2014, p. 20). Cobin (2009) suggested that some investigators may instinctively detect discrepancies at staged crime scenes, such as inconsistencies in the offender's behavior or alibi, and discrepancies between their story and the physical evidence. While Cobin's study focused on murders disguised as hunting accidents, the identified indicators are relevant to broader investigations and include:

- Inconsistent injuries
- Inconsistencies in the circumstances surrounding the incident
- The alleged offender's behavior before and after the incident
- Corroboration of the forensic evidence with the provided story, and
- Potential motives for the murder.

Hazelwood and Napier (2004, pp. 744–759) interviewed 20 law enforcement officers to explore investigation perspectives regarding crime scene staging. They emphasized the importance of understanding the motives behind both the initial crime and the subsequent staging. Motives for the initial crime commonly include greed, anger-revenge, attention-seeking, and game-playing, while motives for staging may include self-preservation and embarrassment-shame (Hazelwood & Napier, 2004, p. 756). Hence, the authors recommended several investigative steps, including conducting a comprehensive scene review, studying victimology, documenting staging indicators, identifying motives for both the murder and staging, and determining who would benefit from the original crime and the subsequent staging.

Pettler (2015, p. 160) suggested that investigating officers prioritize victimology and ask relevant questions to gain insight into the victim's relationships and circumstances. Offenders are increasingly sophisticated in their attempts to deceive investigators, often motivated by a desire to evade detection during the initial investigation (Chancellor & Graham, 2014, p. 24). Bitton and Dayan (2019, p. 1056) proposed that improved tools and criteria in femicide cases could reduce concealment and misidentification. The authors identified signs indicating possible staging, such as the victim's young age and good health, discrepancies at the crime scene, evidence of domestic violence, and the victim being found deceased by an intimate partner (Bitton & Dayan, 2019, pp. 1066–1067). Investigative teams should focus on victimology and pay specific attention to the victim's body, as signs of staging often suggest an intimate partner as the offender (Schlesinger et al., 2012, p. 44). Missing person reports should consider the possibility of verbal staging, guiding investigations accordingly.

Dayan and Bitton (2022) suggested policy and practical implications, including striving for gender-sensitive reviews of women's causes of death and redefining criminal suspicion to encompass a broader range of circumstances. Pettler (2015, p. 156) stressed the importance of police officials being familiar with signs of intimate partner violence to recognize indicators at crime scenes and ask relevant questions about the victim and offender's history.

## Method

This empirical study employs a qualitative research method to investigate the phenomenon of staging of intimate partner murders within the context of Gauteng. Unlike quantitative research, which emphasizes numerical data and statistical measurements, qualitative research focuses on exploring the depth and complexity of social phenomena (Du Plooy-Cilliers et al., 2021, p. 199). By adopting this framework, the study aimed to capture the intricate dynamics involved in staging such crimes, including the motivations, emotions, and perceptions that quantitative tools might overlook. It seeks to uncover meanings, patterns, and insights that are often missed by rigid metrics or predefined variables (Fouché et al., 2021, p. 40). Creswell and Creswell (2017, p. 32) elucidate that qualitative research seeks to understand and interpret the nuances of social phenomena, often through in-depth engagement with individuals or groups. Schwandt and Gates (2017, p. 341), as cited by Fouché et al. (2021, p. 303), discuss the descriptive nature of case studies, highlighting their ability to provide detailed insights into specific phenomena. Du Plooy-Cilliers et al. (2021, p. 209) state that an exploratory case study involves the rigorous description of a case within the broader context to understand the nature and an attempt to explain the circumstances. Given the relatively unexplored nature of staging in intimate partner homicides in South Africa, this study adopted an exploratory-descriptive approach, drawing insights from real-life cases to inform its findings. To conduct the study, ethical clearance was obtained from the university's and SAPS’ ethics review boards.

Qualitative research often employs purposive sampling, a method that allows for targeted selection based on specific criteria rather than random selection (Fouché et al., 2021, p. 382). Purposive sampling was utilized to select cases of intimate partner murders exhibiting characteristics of crime scene staging within the specified geographic and temporal parameters. For this study, 18 concluded intimate partner homicide cases were purposively selected. To be included, cases had to meet the following criteria: the incident must have occurred within the Gauteng province; the case had to be finalized in court, with a clear outcome or verdict; and the homicide must have involved individuals in an intimate relationship. The specification of “intimate relationship” was not limited to marital relationships but included any form of romantic or cohabiting partnership. The cases which had elements of crime scene staging were selected. The selection involved assessing the presence or absence of crime scene staging behaviors such as concealment, as well as less visible forms such as planning, manipulation of evidence, falsifying the narrative of the death to police or others, and obstructing the criminal investigation such as by destroying evidence, creating a false alibi or misleading investigators about the victim's movements or whereabouts (McLachlan & Ferguson, 2024, p. 5). This deliberate selection process ensured that the chosen cases were most relevant to the research objectives, maximizing the depth of insight gained from the analysis.

Data collection involved semistructured interviews with the participants, investigating officers, providing an opportunity to gather rich, contextual information about each case. They had the opportunity to reflect on their case notes/dockets during the interviews; however, those were not analyzed in this study. Informed consent was obtained from each participant prior to the commencement of the interviews. The purpose of the research was clearly explained, and all relevant ethical considerations were addressed. The participants consented that the interviews could be recorded and later transcribed. Interview schedules were meticulously prepared in advance to guide discussions, covering various aspects such as the extent of crime scene staging, crime scene staging variables (modus operandi, weapons used, victimology, and offender profiles), and detection. By employing semistructured interviews, the researcher could maintain flexibility, and include direct quotes from the participants, while ensuring that all relevant topics were adequately addressed.

Transcribed interview data were meticulously organized and coded for analysis using Excel spreadsheets. This coding process involved identifying key themes and patterns within the data, allowing for a systematic exploration of the research questions. Themes and insights derived from the analysis were then interpreted and presented in a structured manner, enabling the researcher to draw meaningful conclusions and develop relevant recommendations. By employing content analysis on the transcribed interviews, the overt and covert themes could be properly explored (Du Plooy-Cilliers et al., 2021, p. 269). The themes that emerged included insights into the experiences of each investigating officer; the demographics and locations of the homicides; detailed information about the victims, offenders, and the nature of their intimate relationships; specific details surrounding the homicides; the staging behaviors observed; and how such cases were identified and detected. The data could then be interpretated to provide socially constructed arguments, later presented in a unique contextualized meaning (Du Plooy-Cilliers et al., 2021, p. 268).

Overall, the qualitative research approach adopted in this study offered a comprehensive and nuanced understanding of crime scene staging in intimate partner murders within Gauteng, South Africa. By engaging directly with investigating officers and analyzing real-life cases, the study aimed to contribute valuable insights to the field, potentially informing future investigative practices and policy development within law enforcement agencies. This approach is particularly suited to this topic, as it involves close interaction between researchers and participants, recognizing that human experiences are subjective and shaped by unique sociocultural contexts, and best understood through open-ended exploration.

## Findings

### A Working South African Definition

To appreciate how crime scene staging in intimate partner homicide can be better detected through context-specific understanding it was essential to examine how this phenomenon is currently perceived by those at the frontline of investigations. This was also necessitated by the scarcity of formal frameworks addressing staging within the South African criminal justice context. This is also needed in laying the groundwork for a localized conceptualization that can inform both practice and policy aimed at improving detection. Thus, the study sought to develop a working definition based on the lived investigative experiences of SAPS officers. Accordingly, investigating officers were asked to explain their understanding of crime scene staging. While four officers had not previously encountered the term, they quickly recognized its meaning once it was explained and were able to recall cases they had worked on that involved staging behaviors. This indicated that it was the terminology, not the phenomenon, that was unfamiliar to them.

The remaining officers provided definitions that fell into three broad categories:

- Offender-focused understanding: Five officers described staging in terms of the offender's behavior and intent. For example, one officer noted, “He pretends he was not on the crime scene and even assists in the investigation,” while another mentioned, “The perpetrator misdirects the way the crime looks.”
- Crime scene-focused understanding: Eight officers focused on the manipulation of the physical scene. They described actions such as “making the crime scene suit his alibi,” and “deliberate tampering with the scene to derail the investigation.”
- Crime-focused understanding: Two officers emphasized attempts to disguise the type of crime committed. One explained, “Making a crime look like another,” while another said, “Make murder look like a suicide.”

These responses highlight that while terminology may vary, many investigators have encountered crime scene staging in practice, recognizing it through the deliberate manipulation of evidence to mislead investigations.

### Crime Scene Staging Variables

In the analysis of the variables associated with staged intimate partner femicide cases in South Africa, it was found that most murders were staged to appear as either committed by an unknown person (four cases) or as suicides (four cases). Following closely were murders staged as sexual assaults (three cases) and botched robbery attempts (three cases). Interestingly, two cases involved intimate partner homicide staged as ritualistic killings, a higher occurrence than anticipated due to South Africa's diverse cultural influences. Conversely, staging methods like accidents or arson were less common, each occurring only once. Among the 18 cases analyzed, various forms of verbal staging were observed, ranging from falsely claiming the victim left with an unknown person to suggesting accidental deaths or asserting self-defense.

Temporal analysis revealed a pattern where most victims were murdered or discovered on weekends (Friday to Sunday), with six cases occurring on Saturdays, three on Friday, and three on Sunday. Late-night to early-morning periods were prevalent times for incidents, followed by mid to late mornings and afternoons. Regarding crime scene characteristics, victims were found in various states of dress, with some fully (4) or partially naked (2), while others were fully clothed (8). Notably, offenders redressed victims in two cases. In most cases (10), no weapon was found at the scene, while others involved firearms, knives, or unconventional items. Strangulation was the most common cause of death (7), followed by gunshot wounds (3), stab wounds (3), blunt force injuries (2), and burn wounds (1).

Most offenders were males, primarily African (13), followed by Caucasian (3), colored (1), and Indian (1). Offenders often aimed to distance themselves from the victim with various alibis or excuses, such as claiming the victim left voluntarily or alleging accidents or suicide. The participants noted specific verbal staging attempts that did not coincide with forensic evidence: “He said that he thought there was an intruder,” “An intruder came and shot him and his wife and ran away,” “He left his girlfriend safely at the house,” “An unknown person came into the house,” “She was too drunk, boiled water and burned herself.”

Seventeen offenders were found guilty, with sentencing varying from life imprisonment to less than 10 years. Conviction rates were high, but sentencing was not consistently stringent. Victim demographics revealed that the majority were African females, primarily between 19 and 29 years old. Most cases involved intimate partner relationships, with varying durations and histories of domestic abuse. Overall, this study provides valuable insights into the staging of intimate partner homicides, highlighting patterns, characteristics, and legal outcomes within the South African context.

### The Prevalence Perceptions of Staging in Gauteng

The investigating officers indicated they had either one to five such cases (9) or more than 10 cases (8). This variability may be linked to their interpretation and understanding of crime scene staging, which likely affected the outcome. From their responses, it can be inferred that staging is either very prominent or not prominent at all. The correlation between years in service and the number of investigated staged murder cases could not be established, as all officers had more than 5 years’ service.

During the analysis of the year distribution, it was noted that there are certain years in which staging becomes more prevalent, as shown in Table 1.

**Table 1.** Year Prevalence of Crime Scene Staging (SAPS, 2023).

Year	Number of cases	%
2011	1	5.55
2012	1	5.55
2013	1	5.55
2014	3	16.66
2015	3	16.66
2016	0	0.00
2017	1	5.55
2018	1	5.55
2019	2	11.11
2020	4	22.22
2021	1	5.55
Total	18	100

**Figure 1.**
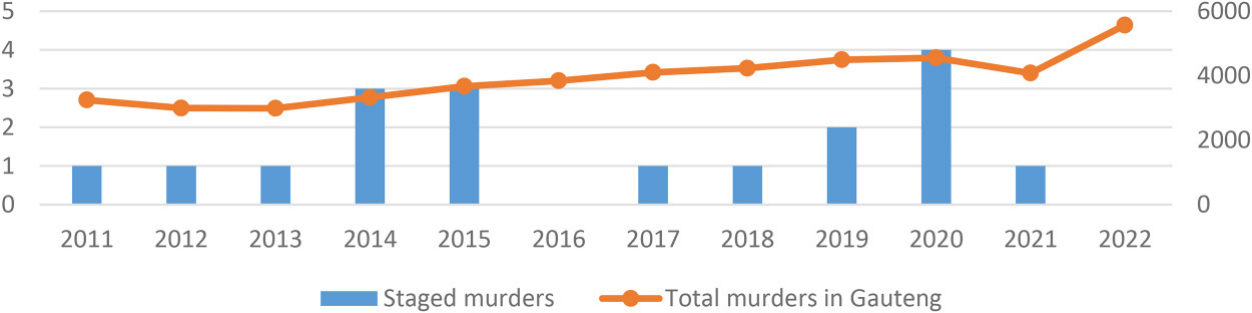
Linear relation between staged crime scenes and femicide cases (SAPS, 2023).

There were more cases in 2014 and 2015, and later in 2019 and 2020. There appears to be a linear relation to the years when femicide was more prominent, as indicated in Figure 1.

### The Role of the SAPS

The participants indicated that first responders have various responsibilities at the crime scene, with many agreeing that they have more than one duty. Most participants cited the first responsibility of first responders as establishing the safety of the scene and cordoning it off to preserve its integrity (12). Following this, their duties include gathering and safeguarding evidence (7), calling relevant stakeholders (5), identifying witnesses (3), and determining the victim's identity (1). Additionally, there was a consensus among three participants that first responders should refrain from disturbing the scene and wait for detectives to conduct the investigation. One participant noted that, while first responders are assigned various duties as mandated by the SAPS, they are not responsible for detecting crime scene staging.

Participants highlighted the crucial role of Local Criminal Record Centre (LCRC) members and forensic experts in detecting crime scene staging. Taking proper crime scene photographs was deemed the most important task (11), followed by collecting and analyzing forensic evidence such as blood spatter, ballistic evidence, fingerprints, and DNA (9). Two participants mentioned the collection and analysis of physical exhibits as well. Six participants emphasized the role of LCRC and forensic experts in confirming or refuting the investigating officer's suspicions and correctly identifying the type of crime. However, participants expressed concerns about the lack of contextual understanding among LCRC members and forensic experts, leading to hasty processing of the crime scene and potential oversight of important forensic evidence or poor-quality crime scene photographs. Most participants agreed that investigating officers should actively participate in processing the crime scene alongside LCRC members and forensic experts (11). They should analyze all facts comprehensively.

### The Detection of Crime Scene Staging

In this study, it was important to establish how crime scene staging was detected in the cases and whether it was easily detected or not. Most participants (7) noted various indications suggesting staging. Among them, most highlighted that the incident deviated from typical characteristics expected at such crime scenes. Additionally, some participants (4) pointed out the absence of forced entry, while others (3) identified attempted concealment of the victim's identity as a clear staging indication. Less frequently noted indicators included a disorganized crime scene (1), lack of forensic evidence supporting the scene (1), and delayed initiation of a missing person's case (1). One participant (1) initially found no staging indications upon attending the crime scene.

Participants expressed confidence that, in most cases, the correct suspect was identified and subsequently arrested, with the majority (17) agreeing on this point. However, three participants (3) were less confident. One participant mentioned successful arrest and conviction of a suspect in cases they were personally involved in and where they physically attended the crime scene. When asked about the accuracy of prosecutions and convictions, the majority (17) concurred that convictions were obtained, although some participants (6) acknowledged the occurrence of wrongful arrests. All participants acknowledged the invaluable role of the SAPS in detecting crime scene staging. Some participants emphasized that the SAPS are the primary actors in this regard, highlighting the significance of early detection rather than relying solely on the justice department or later stages of the criminal justice process.

Regarding the accuracy of findings by the inquest court, most participants (16) believed them to be mostly accurate. However, one participant expressed skepticism, suggesting that accuracy could improve if suspects or persons of interest were required to testify formally in an inquest. Another participant echoed this sentiment, emphasizing that findings from formal inquests are generally more accurate than those from informal proceedings.

## Discussion

In this study, it was crucial to discern how crime scene staging variables in South Africa compared to international ones, and whether the same detection techniques could subsequently be applied. While most investigating officers demonstrated a grasp of the concept, there were variations in their understanding, with some requiring clarification during the interviews. The results revealed that most crimes were staged to appear as suicides or homicides committed by an unknown third party. This trend could be attributed to the high overall murder rate in the province, which perpetrators exploited, especially in intimate partner cases. This can be better explained by employing the rational choice theory in that they selected the type of murder meticulously and used the high murder rate in the province as a disguise to avoid being suspected of the crime. The prevalence of staged suicides aligns with findings from previous studies (Chancellor & Graham, 2014; Dayan & Bitton, 2022; Ferguson, 2021; Pettler, 2015).

Verbal staging was a common feature in most cases, reflecting a continuation of deception by offenders at the crime scenes, which aligns with international findings (Ferguson, 2021; McLachlan & Ferguson, 2024). This verbal staging, often limited by offenders’ imaginations and intellectual capabilities, could easily be contradicted by physical evidence, similar to findings from Dayan and Bitton (2022). The staging behavior, particularly the verbal staging, could be seen as a continuation of the rational choice taken to avoid detection and maximize the rewards, however varied (Ferguson, 2021). A recurring theme was the correlation between the type of crime staged and the most prevalent murder type in each geographical area. The homicides that occurred in areas where the murder-robbery rate was very high, had crime scenes staged accordingly, etc.

Additionally, the study identified a notable concentration of murders and staging occurring on weekends. While this may possibly be linked to increased alcohol consumption and heightened contributory factors such as low self-esteem and aggressive behavior (Bezuidenhout, 2023, p. 285), it may also indicate an exploitation of situational opportunity structure by the offenders. In other words, offenders appear to commit these acts during times when explanations like intoxication, argument escalation, or absence of witnesses may be more believable or less scrutinized. This, again, aligns with a rational assessment of when to act with minimized risk of being found out, as the motives for staging often include self-preservation (Hazelwood & Napier, 2004, p. 756).

Regarding crime scene locations, most murders occurred within or near the residences of the victims or offenders, consistent with existing literature and theoretically based that victim selection and location of an offender, which further provided new insights into the primary and secondary scenes. The study found that when the primary scene was the offender's residence, a secondary scene often involved the disposal of the victim's body elsewhere.

The study further shed light on the methods used by offenders, with strangulation being the most common cause of death, followed by gunshot wounds, sharp and blunt force injuries, and burn wounds. Notably, excessive injuries, or “overkill,” were observed in many cases, reflecting expressive aggression commonly associated with intimate partner murders, coinciding with a study by Martins (2019). The instrumental logic of detection avoidance as well as the emotional logic of guilt management or legacy control also underscores the dynamics of rational decision making. Relatedly, another significant finding was the phenomenon of “undoing,” where offenders attempted to psychologically reverse the crime by redressing victims or altering the crime scene. This behavior, along with the removal of items from the scene, reflected characteristics of both cleaner and concealer typologies in offender behavior (Pettler, 2015).

In terms of offender demographics, the study revealed a predominance of African male offenders in their 20s to 30s, with few in law enforcement roles. This differed from previous studies but highlighted the importance of considering local demographics and social dynamics. Femicide remains a troubling phenomenon in South Africa, particularly evident in analyses of intimate partner murders within Gauteng province. Research indicates a stark gender disparity, with femicide far outweighing instances where males are victims, a trend echoed in the findings of Abrahams et al. (2013, p. 3). Investigating officers often assert that women in murder cases are more likely to be killed by acquaintances, aligning with Hazelwood and Napier's (2004, p. 758) observations, primarily within intimate partner/ex-partner relationships. Notably, these relationships predominantly involve individuals of the same racial groups, commonly characterized as boyfriend–girlfriend or marital unions, with over 50% lasting longer than a year and all being heterosexual in nature.

A striking revelation emerges from these studies: a lack of reported prior domestic violence in most cases. This aligns with Turvey's assertion that staging serves as a means for offenders to dissociate themselves from their victims and subsequent crimes (Turvey, 2000). Consequently, investigating officers often scrutinize intimate partners as potential suspects, a perspective supported by Pettler (2015, p. 82), who emphasizes the centrality of interpersonal relationships in staging analyses. Given that strangers typically lack motive for primary staging, the presence of an intimate partner relationship emerges as a pivotal determinant in staged crime scenes (Ferguson & McLachlan, 2023).

The study emphasized the importance of victim-focused investigations and a team-based approach to crime scene management, with experienced investigating officers playing a crucial role in identifying staging. It also advocated for improved lie detection techniques, the utilization of trained professionals like criminologists, and the recruitment of reliable informers to enhance investigations. The following 10-point plan was derived from international detection practices with the inputs from the participants to enhance detection and improve investigation skills in staged intimate partner homicide cases:

1. A crime scene-centered approach

Most participants concurred that adopting a crime-scene-centric approach is imperative, particularly in the investigation of murders. They emphasized the significance of the crime scene as a repository of crucial clues and insights into the unfolding events. It was underscored that perpetrators inevitably leave traces or remove items, namely forensic evidence, which can offer invaluable leads. Bezuidenhout (2011, p. 405) highlights the pioneering work of Hans Gross, who, in 1924, authored the first treatise on crime scene staging, advocating for the application of scientific methodologies in criminal investigations. Gross (1924) regarded every crime scene as a scientific puzzle demanding resolution. Building upon Gross's principles, Edmond Locard, in 1910, demonstrated their practical application in crime laboratories. Locard's Exchange Principle posits that when a criminal interacts with a person or object, there is a transfer of evidence, enabling the linkage of suspects to crime scenes. It was emphasized that even seemingly trivial details on the crime scene can yield significant insights upon closer examination. The responsibility for thorough crime scene analysis was noted to commence with first responders, who play a pivotal role in the investigative process. Their initial approach sets the tone for subsequent procedures, necessitating adherence to proper crime scene management protocols from the outset. Despite efforts at crime scene reconstruction, participants lamented the occasional dearth of vital information, citing the removal of victims from the scene as a hindrance to achieving a comprehensive understanding. Consensus was reached on the pivotal role of the victim, advocating for prioritizing victim-centered approaches in crime scene investigations.

2. A victim-focused investigation

Various scholarship, including that by Abrahams et al. (2013), Brodie et al. (2023); and Centre for the Study of Violence Reconciliation, CSVR (2016) have pointed out that a significant proportion of women's murders are perpetrated by individuals known to them. Although target hardening initiatives have been suggested and implemented by the authorities, as stipulated as a factor in the rational choice theory (Morgan et al., 2012, p. 53), the prevention of femicide remains problematic. Consequently, prioritizing the victim and adopting an outward investigative approach, beginning with the victim's inner circle, is crucial. Bitton and Dayan (2019, p. 1064) advocate for a criminological examination of women's fatalities to ascertain if the circumstances align with initial crime scene perceptions.

Interviewing the victim's immediate family, friends, and acquaintances on-site and obtaining their statements is essential. Given that all victims in this study were female, it is imperative to handle cases involving female victims with meticulous attention to detail, as Bitton and Dayan (2019, p. 1066) delineate six indicators of suspicion or criminality:

1. Unexpected death of a seemingly healthy individual with no prior medical history indicating otherwise.
2. Suicide by a woman, particularly if she is a mother.
3. Indications of a desire to terminate a relationship or evidence of infidelity by one of the intimate partners.
4. History of domestic violence, whether documented in formal cases or attested to by relatives or acquaintances.
5. Death of a woman in her own residence.
6. Discovery of the woman's body by her intimate partner or the partner reporting her as missing.

3. Team-based crime scene management.

Various stakeholders and participants involved in crime scenes may hold diverse perspectives and opinions. Acknowledging and incorporating these viewpoints is crucial, particularly if the lead investigating officer overlooks any crucial details. Consensus among participants was reached on the collaborative nature of processing, investigating, and managing a crime scene, emphasizing that teamwork yields superior outcomes. Effective communication and consultation with all role players and stakeholders are essential, ensuring comprehensive liaison and coordination throughout the investigative process.

4. Diligent processing of the scene

Participants emphasized that the rushed processing of crime scenes, stemming from high workloads and negligence among role players, often leads to the oversight of valuable evidence. They advocated for a more meticulous approach to scene processing, urging against haste. While acknowledging the importance of swift response by first responders to preserve forensic evidence, participants stressed the need for subsequent role players to exercise diligence in processing the scene. Investigating officers were advised to allocate sufficient time at the scene, ensuring a controlled and thorough processing procedure.

5. The utilization of experienced investigating officers

During interviews with participants, a recurring observation was the value of experience in managing murder cases and identifying potential crime scene staging. Consequently, it was suggested that only investigating officers with substantial experience in such cases should handle murder crime scenes. These seasoned officers should maintain an open-minded approach, avoiding snap judgments. Bitton and Dayan (2019, p. 1064) emphasize the necessity for objective investigation, devoid of preconceptions. By focusing on the minutiae, experienced officers can discern crucial details. To facilitate knowledge transfer, newer members could accompany experienced officers to scenes for learning opportunities.

Moreover, experienced investigating officers shouldn’t limit their involvement to murder cases alone but should also attend any unnatural death scenes. Every instance of unnatural death warrants consideration as a potential murder scene, underscoring the importance of their expertise in various investigative contexts.

6. Improved lie detection

Detecting crime scene staging relies heavily on the ability to discern deception. Matsumoto et al. (2011) examined videos featuring 10 participants, half of whom were truthful and half deceptive, aiming to distinguish between them by analyzing nonverbal cues and statements. The findings revealed that liars exhibited significantly more nonverbal behaviors inconsistent with the context of their words. Linguistic analysis further highlighted that liars tended to use minimizing and editing adverbs, along with alterations in nouns and verbs. Combining inconsistent facial expressions with statement analysis enabled the accurate classification of 90% of the deceptive individuals. Consequently, it's imperative to recognize these nonverbal cues.

Moreover, physical evidence at the crime scene should either corroborate or contradict statements made by potential persons of interest. It's essential for all role players at the scene to refrain from automatically accepting statements at face value, given the potential for deception.

7. Utilization of trained professionals

Established in 1996, the Investigating Psychology Section (IPS) operates as a national unit, extending its assistance to cases not only within the country but also in neighboring nations. Tasked with addressing psychologically motivated crimes, the IPS focuses on offenses where financial gain isn’t the primary motive. These include but are not limited to serial murder, mutimurder, serial rape, sexual murder, and intimate partner murder. Its support encompasses a range of services aimed at aiding investigating officers, such as investigative assistance, detective training, and research.

The unit's investigative support comprises consultations with officers, crime scene and behavioral analysis, witness interviews, and expert testimony provision in court. Trained criminologists play a pivotal role, particularly in applying behavioral analysis techniques at crime scenes. In the context of crime scene staging, criminologists analyze various aspects to deduce the motive behind the murder and subsequently profile the type of offender likely responsible.

8. The forensic pathologist and the crime scene

Participants stressed the importance of having a forensic pathologist present at crime scenes to gain insights into the body's positioning, the surrounding environment, and the nature of the wounds. However, logistical constraints often hinder this practice due to time and resource limitations. A potential solution proposed involves training forensic pathology assistants, responsible for transporting victims’ bodies to the mortuary, to provide comprehensive reports to the pathologist. These assistants would be equipped to capture photographs of the victim at the scene, allowing the pathologist to visualize the conditions encountered during the postmortem examination. Coupled with the investigating officer's attendance at the postmortem examination, this collaboration could prove invaluable in piecing together crucial details of the case.

9. Recruitment of reliable informers

Minnaar (2011, p. 90) underscores the significance of informers as valuable sources of information for investigating officers, particularly in cases where crimes go unreported, or community members are hesitant to provide statements. When effectively managed, informers can offer invaluable insights. In instances of intimate partner murders, prior instances of domestic abuse are often undisclosed, even to close acquaintances. However, there may be occasions where individuals within the victim's inner circle were aware of the abuse or observed warning signs.

Given that previous domestic abuse aligns with indicators of suspicion as outlined by Bitton and Dayan (2019, p. 1066), such information proves immensely beneficial in discerning crime scene staging. However, due to distrust in law enforcement, community members may be reluctant to engage with police officials but may be more willing to share information with someone from within their community. Convicted crime scene stagers (Schlesinger et al., 2012) could be an invaluable source of information.

10. Research, training, and awareness

Many participants, despite working with staged crime scenes, were unfamiliar with the term “staging.” Conducting research on this subject could equip them with the necessary knowledge to recognize staged scenes. IPS could play a pivotal role in conducting research and providing training to investigating officers. This research shouldn’t be confined to South African sources but should encompass international literature to enhance the understanding and handling of staged crimes.

Training and raising awareness will acquaint role players with the concept of staging, enabling them to identify it effectively at crime scenes. Specialized training holds the potential to facilitate the apprehension of the correct offenders and improve prosecution outcomes, subsequently reducing civil claims against the police and the criminal justice system. Access to such training and awareness should extend beyond investigating officers to include all personnel involved in crime scenes, including first responders.

Moreover, equipping investigating officers with specialized training in linguistic profiling and statement analysis, along with techniques for detecting deception through nonverbal cues, would significantly enhance their ability to identify inconsistencies and uncover hidden motives. Such training would foster more precise investigations, ensuring that subtle indicators of staging are not overlooked, and justice is effectively pursued.

## Limitations

While this study offers important contributions to the underexplored topic of crime scene staging in South Africa—particularly within the context of intimate partner homicides—it is not without limitations.

Firstly, the study is geographically limited to the Gauteng province, South Africa's most urbanized and economically advanced region. Although efforts were made to include police stations across a diverse range of socioeconomic areas, the findings may not be fully representative of crime scene staging practices in more rural or less-resourced provinces, where investigative capacity, infrastructure, and crime dynamics may differ significantly.

Secondly, the research relied on qualitative data gathered through interviews with 18 investigating officers. While their insights are invaluable, the sample size remains relatively small and purposively selected, which may affect the transferability of the findings. Additionally, the reliance on officers’ recollections and subjective interpretations of past cases introduces the possibility of recall bias, particularly in instances where official documentation on crime scene staging was limited or where the terminology was not explicitly used at the time.

Another limitation is the absence of triangulation with other data sources. The study did not incorporate perspectives from forensic analysts, prosecutors, or psychological experts, nor did it include direct analysis of physical crime scenes or court transcripts.

Furthermore, while the study draws comparisons with international literature and applies rational choice theory to understand offender behavior, the scope of theoretical engagement was limited to a single framework. Broader theoretical triangulation could provide a more nuanced understanding of staging motivations and decision-making processes, as seen in other studies where feminist theory was applied (Ferguson & McLachlan, 2023).

Lastly, the study's focus on heterosexual intimate partner relationships, as reflected in the available cases, means that the findings may not generalize to staging practices in LGBTQIA+ relationships or in cases involving female perpetrators. As such, the research reflects a specific profile of staging that may differ in other relational or cultural contexts.

Despite these limitations, the study represents a foundational step in understanding crime scene staging in South Africa and offers practical implications for investigative practice, policy development, and future academic research.

## Conclusion

This study delved into the nuances of crime scene staging specific to South Africa, particularly focusing on cases within the Gauteng province. Alongside studying the variables associated with staging, the prevalence and detection thereof were explored. It was further investigated whether the concept of staging could be theoretically grounded in rational choice theory. This theory applies in that offenders make rational choices regarding both the crimes they commit and their selection of victims, and it extends to their staging behaviors as well. This approach provided valuable insights into potential overlap with international variables and deficiencies in current investigations, contributing to the development of a 10-point plan through the combination of international detection strategies and inputs from the participants. Although the focus was on detection rather than prevention, improved efforts have the possibility of significant advances in target hardening and risk amplification according to Morgan et al. (2012, p. 53) exploration of crime response factors in the rational choice theory. This can subsequently thwart future offenders.

Similar to prior studies by Ferguson (2015), Pettler (2015), Schlesinger et al. (2012), and Bitton and Dayan (2019), this study primarily examined primary staging, with secondary and tertiary staging not present in sample cases. Contrary to the anticipated prevalence of murders staged as robberies due to South Africa's high incidence of robbery-related crimes (Bezuidenhout, 2023), findings indicated a predominance of murders staged as suicides or perpetrated by unknown individuals.

Notably, this study sought to augment existing literature on staging by incorporating temporal factors to identify common trends. Most murders occurred on weekends, possibly attributed to heightened alcohol and substance consumption, increased social interactions, and disrupted routines. Predominant methods of murder included strangulation, gunshot wounds, blunt force trauma, and sharp force injuries, mirroring findings from Ferguson (2021). The presence of overkill was not unexpected, aligning with Martin'’s (2019) observations on intimate partner murders. The offender profile in this study reflected racial demographics outlined by Cowling (2024), with mostly male offenders in their 20s and 30s, acting alone and rarely affiliated with law enforcement. Victim profiles largely concurred with previous studies, predominantly comprising African females in their late teens and 20s, all involved in intimate relationships with the offenders.

While participants articulated the expected roles of various role players on crime scenes in line with existing legislation and directives, a notable concern emerged regarding a potential oversimplified approach to crime scene management. Detecting crime scene staging was deemed primarily the responsibility of law enforcement, necessitating combined expertise from forensic specialists, investigating officers, and forensic pathologists. However, some participants suggested that proper training and experience would enable investigating officers with a detail-oriented approach to detect staging on-site.

A crucial concern was the infrequent inclusion of obstruction of justice charges in cases involving staged intimate partner murders. This highlights the critical need for police officials to recognize and address staging early in their investigations to ensure justice is served. The developed 10-point plan focuses on equipping police officials with the necessary skills and strategies to identify signs of staging, improve investigative accuracy, and promote timely interventions toward addressing the challenges in the context of South Africa, especially Gauteng.
